# Invasion Ability and Disease Dynamics of Environmentally Growing Opportunistic Pathogens under Outside-Host Competition

**DOI:** 10.1371/journal.pone.0113436

**Published:** 2014-11-21

**Authors:** Ilona Merikanto, Jouni T. Laakso, Veijo Kaitala

**Affiliations:** 1 Department of Biosciences, University of Helsinki, Helsinki, Finland; 2 National Institute for Health and Welfare, Helsinki, Finland; 3 Centre of Excellence in Biological Interactions, University of Jyväskylä, Jyväskylä, Finland; Louisiana State University, United States of America

## Abstract

Most theories of the evolution of virulence concentrate on obligatory host-pathogen relationship. Yet, many pathogens replicate in the environment outside-host where they compete with non-pathogenic forms. Thus, replication and competition in the outside-host environment may have profound influence on the evolution of virulence and disease dynamics. These environmentally growing opportunistic pathogens are also a logical step towards obligatory pathogenicity. Efficient treatment methods against these diseases, such as columnaris disease in fishes, are lacking because of their opportunist nature. We present a novel epidemiological model in which replication and competition in the outside-host environment influences the invasion ability of a novel pathogen. We also analyze the long-term host-pathogen dynamics. Model parameterization is based on the columnaris disease, a bacterial fresh water fish disease that causes major losses in fish farms worldwide. Our model demonstrates that strong competition in the outside-host environment can prevent the invasion of a new environmentally growing opportunist pathogen and long-term disease outbreaks.

## Introduction

Environmentally growing opportunistic pathogens that survive and reproduce in the outside-host environment e.g. as saprotrophs are abundant in nature [1]–[3]. Survival and growth of environmentally growing opportunistic pathogens can thus be completely host-independent. Well-known pathogens, such as *Vibrio cholera*, *Pseudomonas aeruginosa, Legionella pneumophila, Listeria monocytogenes, Cryptococcus neoformans* and many species from *Mycobacterium, Flavobacterium* and *Serratia* genus, are environmentally growing opportunists as within-host growth is more of an alternative reproduction strategy [1], [3]–[14]. Treating or temporarily removing susceptible hosts does not prevent new outbreaks of environmentally growing opportunistic diseases, as has been demonstrated with *V. cholera* [9], [15].

Environmental opportunism may also lead to the evolution of high virulence because host-independent long-term survival can, at least partially, relax the transmission-virulence constraints limiting host-specific obligatory pathogens [16]–[20]. However, the relationship between environmental transmission and virulence can be more complicated than this. We refer to virulence here as the harm caused by the infection, such as increased mortality as well as an inability to reproduce. In nature virulence of pathogens varies from quite harmless to pathogens causing both sterilization and high mortality in hosts. Here we address pathogens that exhibit high costs to their hosts, such as both sterilize and increase mortality of their hosts. An ability to infect host successfully is referred to as pathogenicity. Evolution of high virulence can also be found in pathogens with multiple hosts or in pathogens that are able to survive outside-host environment for long periods e.g. as resting spores otherwise in a passive state [21]–[25]. The difference between these kind of pathogens and environmentally growing opportunists is that the latter also grows host-independently indefinitely by using outside-host resources and also competes with other microbes in the outside-host environment for these resources. Thus selection forces in the outside-host environment, such as competition and predation, are likely to influence the evolution of pathogenicity and virulence in environmentally growing opportunists as well as their disease dynamics [1], [26]–[28]. Environmental opportunism might be beneficial under outside-host competition, as environmentally growing opportunists gain a competitive advantage through within-host growth. For instance, many aquatic and soil bacteria contain virulence factors [1], [29], [30]. They can thus function as environmentally growing opportunists by changing their gene expression. For example, in *P. aeruginosa* the expression of virulence factors is promoted as bacteria densities and competition increase, enabling them to escape the outside-host competition into within-host environment [31]. Strong competition outside the host can also restrict invasion of a new environmentally growing opportunist pathogen if they are inferior competitors in the outside-host environment [1], [11].

It has also been suggested that environmentally growing opportunist pathogens are a pathway to obligatory pathogenicity [1]. Genome reduction is common as free-living microbes adapt to within-cell environment [32]. Therefore, it is possible that the ability to replicate and survive in the outside-host environment might be weakened if essential metabolic pathways related e.g. to saprotrophism are lost in a trade-off to within-cell adaptation. This could promote shifting from environmental opportunism to obligatory pathogenicity with time. However, there is no theoretical framework of how environmentally growing opportunist pathogenicity develops in the first place. Models of the evolution and disease dynamics of environmentally growing opportunistic pathogens are needed in order to predict and manage novel diseases and their outbreaks.

Merikanto et al. [15] presented a model for coupling outside-host growth with long-term host-pathogen dynamics. Godfray et al. [33] also considered both the influence of outside-host growth and outside-host competition on disease dynamics. However, Godfray et al. only considered short-term disease dynamics and did not address the evolution of environmentally growing opportunistic pathogenicity. Other models that have considered evolution of virulence of environmentally transmitted pathogens have not addressed pathogens that also grow outside-host [34], [35], [36].

Coincidental virulence theory proposes that pathogenicity is promoted if new traits that benefit survival or growth in the outside-host are also coincidentally virulence factors [3], [16], [17]. In within-host context, multiple infections enhance *P. aeruginosa* toxin production, which helps this environmentally growing opportunist pathogen compete against other pathogenic strains within-host but also leads to higher virulence of *P. aeruginosa* [34]. It is thus possible that coincidental evolution of pathogenicity occurs due to microbial competition, at least in the within-host context. Yet, empirical data has shown that there is often a trade-off between survival and growth in the outside-host environment and virulence [11], [28], [35]. These trade-offs between survival or growth in the outside-host environment and virulence explain why being specialized in living only in the outside-host environment as a free-living microbe or within-host as an obligatory pathogen could be beneficial and why not all the microbes are environmentally growing opportunist pathogens. While these trade-offs might not exist in every case, they do concern many environmentally growing opportunist pathogens. Our aim is to study how have these pathogens been able to invade and whether outside-host competition could prevent disease outbreaks also in established pathogen populations.

Here, we introduce a model for environmentally growing opportunistic disease that considers the presence of a superior non-pathogenic competitor in the outside-host environment. We analyze how a new environmentally growing opportunist pathogen strain is able to survive and replicate in the outside-host environment, where it faces competition and gains an advantage from within-host growth. We also analyze how the outside-host competition influences long-term disease dynamics of environmentally growing opportunist. Parameterization of the model is based on columnaris disease found in freshwater fishes all over the world. This disease caused by saprotrophic bacterium *Flavobacterium columnare* is a major hazard in fish farms [12], [36], [37].

## Methods

### Model of host-pathogen-competitor interaction

We consider a deterministic continuous time model combining environmentally growing opportunist pathogen-host interaction and outside-host competition. The model combines SI dynamics based on model G of Anderson and May [38], pathogen outside-host growth and Lotka-Volterra competition to describe changes in time (*t*) in the densities of susceptible hosts (*S*), infected hosts (*I*), and both pathogens (*P*) and non-pathogenic strain (*B*) in the environment outside-host:
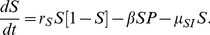
(1)

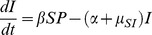
(2)

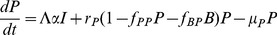
(3)

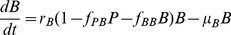
(4)
Equation 1 describes the density change of the susceptible host population (*S*) in time (*t*). Susceptible host population increases logistically in a density-dependent way depending on the growth rate *r_S_.* Host carrying capacity is assumed equal to 1. Susceptible host population is suppressed as they die at rate μ*_SI_* and are infected at rate β. This environmental transmission rate β determines the increase of infected host population (eqn. 2) depending on population sizes of *S* and *P.* As direct transmission between hosts does not influence the disease dynamics drastically, we left this out of our model for simplicity (Supplement S1, Figure S1). We assume that infection sterilizes infected hosts, as is the case in columnaris disease. Also, as fishes infected by columnaris disease generally cease feeding [37], we have dismissed resource competition between infected and susceptible hosts from the model. However, we also compared how possible resource competition between healthy and infected hosts would affect the disease dynamics (model in Supplement S2). For simplicity, host carrying capacity (G) was set to 1. Infected host population is suppressed as they die of infection at a rate α (indicating virulence) or due to other causes at the same rate as *S* (μ*_SI_*)_._

Pathogen population outside-host (eqn. 3) increases as new pathogens are released at rate Λ (referred also as burst size) [22] from infected hosts as they die due to infection. For simplicity, we assume pathogen release only during death, as the release from living hosts is considerably smaller as compared to release rate from dead hosts regarding columnaris disease, on which we are basing our parameterization [12]. Saprotrophic environmentally growing opportunist pathogens, such as *F. columnaris* or *Serratia marcescens*, also use the host for within-host growth after the host is dead. Therefore release rate can be kept as a constant because in saprotrophic environmentally growing opportunist pathogens the release rate is independent of the infection time, as the infection time does not limit the release rate.

Pathogens also increase logistically with an outside-host growth rate *r_P_*, where the density effect of the pathogen's own population is *f_PP_* and the weight of non-pathogenic population to pathogen growth *f_BP_* (referred as also the competition coefficient). Pathogens die at rate μ*_P_.* Non-pathogenic population (eqn. 4) increases logistically with growth rate *r_B_*, where density dependence is of the same form as for the pathogenic population, now the weights being *f_BB_* (density effect of the own population) and *f_PB_* (density effect of the pathogenic population). Non-pathogens die at rate μ*_B_*.

#### Model versions

In the Supplementary section we present versions of our model that are applicable for specific cases of environmentally growing opportunist pathogens. In Supplement S1, we analyze a model that also includes direct transmission of disease between hosts into account. This might occur, for instance, in some fungal diseases where direct contact between hosts spreads skin infection. In Supplement S2 we present a model where healthy and infected hosts compete for same resources. In Supplement S3, we analyze the case where recovery from infection is possible. This may be more suitable for environmentally growing opportunist diseases where host immune system is able to overcome the disease with full recovery of the infected host. In Supplement S4, we address the situation where novel pathogens are released continuously from the infected hosts as compared to release only upon host death. Finally, in Supplement S5, we analyze the invasion of benign environmentally growing opportunists that sterilize their hosts but do not cause any extra mortality due to the infection.

### Parameterization of the model

Parameter values used in the invasion and stability analyses were picked to present a large range of plausible biological values for environmentally growing opportunist pathogens and their potential hosts, especially regarding columnaris disease. The parameter values used are given in Table 1 for invasion analyses and in Table 2 for stability analyses. The parameter values were set to present realistic values when one time unit corresponds to one day [15). For simplicity, we assume in all the analyses *f_PP_* = *f_BB_*. Parameter values for *f_PP_* and *f_BB_* were selected so that the carrying capacities of *F. columnare* strains are comparable to those in the absence of the host.

**Table 1:** Parameter values per time unit (one day) used in the invasion analysis.

*Parameter*	*Explanation of the parameter*	*Parameter values (day^−1^)*
*r_S_*	Susceptible host growth rate	0.01 in Fig. 2a-e or 0.01–0.5 in 2f
*r_P_*	Pathogen growth rate outside-host	0.05 in Fig. 2a–d and 1f or 0–4 in 2e
*r_B_*	Non-pathogenic strain growth rate	5
*μ_SI_*	Mortality of the susceptible and infected hosts due to other reasons than infection	10^−3^
α	Virulence (Mortality of the infected hosts due to infection)	0.1 in Fig. 2a–b and in 2d–f or 0–0.1 in 2c
*μ_P_*	Pathogen mortality outside-host	0.1 in Fig. 2a–c Fig. 2e–f or 0–0.8 in 2d
*μ_B_*	Non-pathogenic strain mortality outside-host	0.1
β	Pathogen transmission rate to susceptible hosts from environment	10^−5^ in Fig. 2b–f or 0–10^−5^ in 2a
Λ	Pathogen release rate from infected hosts when they die	10^5^ Fig. 2a and 2c–f or 0–10^5^ in 2b
*f_PP_*	Negative influence of pathogen population density on its growth	10^−5^
*f_BB_*	Negative influence of non-pathogen population density on its growth	10^−5^
*f_PB_*	Negative influence of pathogen population density on non-pathogenic strain growth	10^−5^
*f_BP_*	Negative influence of non-pathogen population density on pathogen population growth (Competition coefficient)	0–10^−4^ or 0–2×10^−4^ in Fig. 2c

**Table 2:** Parameter values per time unit (one day) used in the stability analysis.

*Parameter*	*Explanation of the parameter*	*Parameter values (day^−1^)*
*r_S_*	Susceptible host growth rate	0.01 in fig. 1b and 4a–b, 1 in fig. 1a and 4c and 0–2 in fig. 3a–c
*r_P_*	Pathogen growth rate outside-host	0.001–0.5 in fig. 2, 0.05 in fig. 4a–b, 3a and 3b and 0.5 in fig. 4c
*r_B_*	Non-pathogenic strain growth rate	1
*μ_SI_*	Mortality of the susceptible and infected hosts due to other reasons than infection	10^−3^
α	Virulence (Mortality of the infected hosts due to infection)	0.1
*μ_P_*	Pathogen mortality outside-host	0.1
*μ_B_*	Non-pathogenic strain mortality outside-host	0.1
β	Pathogen transmission rate to susceptible hosts from environment	10^−5^
Λ	Pathogen release rate from infected hosts when they die	10^5^
*f_PP_*	Negative influence of pathogen population density on its growth	10^−5^
*f_BB_*	Negative influence of non-pathogen population density on its growth	10^−5^
*f_PB_*	Negative influence of pathogen population density on non-pathogenic strain growth	10^−7^ in fig. 3a-b and in fig. 4b, otherwise 10^−5^
*f_BP_*	Negative influence of non-pathogen population density on pathogen population growth (Competition coefficient)	0–10^−5^ in fig. 4a, 10^−7^–10^−4^ in fig. 4b, 10^−8^–10^−4^ in fig. 4c, and 10^−4^ in fig. 3b. Otherwise 10^−5^

Infectiveness is usually assumed to be lower in environmentally growing opportunist pathogens regarding other than immunocompromised hosts as compared to obligatory [44]. The environmental transmission rate (β) for the pathogen was therefore kept low.

Susceptible host growth rates (*r_S_*) and mortality due to infection (α) corresponds to those seen in multicellular hosts of environmentally growing opportunistic pathogens of *F.columnare* and *S. marcescens* [15]. Mortality of the hosts due to other reasons than infection (μ*_SI_*) was given a lower value than α, as seen in nature in many cases [38].

#### Parameterization of outside-host competition

We assume that the non-pathogenic strain is a superior competitor in the outside-host environment. This could result from trade-offs between capability to invade and live within-host, and the efficiency of using outside-host resources for growth or survival [1], as has been seen in the case of *L. monocytogenes* populations that differ in virulence [11]. Also, empirical data has shown that in the case of *S. marcescens* there is a trade-off between virulence and the ability to defend against predation in the outside-host environment [28], [35]. Switching to within-host environment commonly results in genome reduction. Free-living bacteria have a larger genome than environmentally growing opportunists and gene loss increases as bacteria became obligate to the within-host environment [32]. As the outside-host environment is not as stable or consist more of antagonistic ecological interactions than the within-host environment, it is likely that genome reduction limits the ability to utilize variable resources in the outside-host environments due to loss of metabolic functions. Thus it is possible that environmentally growing opportunist pathogens are less equipped to face multiple challenges in the outside-host environment as compared to non-pathogenic strains once their genome has been reduced [1], or once they allocate energy to expressing genes that enable virulence as in the case of *L. monocytogenes* [11]. Therefore, in the parameterization, pathogen growth in the outside-host environment (*r_P_*) was either assumed lower or pathogen mortality (μ*_P_*) as assumed higher than growth (*r_B_*) and mortality (μ*_B_*) of non-pathogen. Pathogen mortality (μ*_P_*) varies in the range of observed mortality values measured in aquatic bacteria [39], [40]. Non-pathogen mortality (μ*_B_*) was standardized to the lower value of the pathogen mortality range according to the assumption that it is a better competitor than the pathogen. Non-pathogen and pathogen growth rates (*r_B_* and *r_P_*, respectively) correspond to average growth rates observed in bacteria [41], [42], [43]. Pathogen growth varies in lower values than standardized growth rate in non-pathogen, again according to the assumption that the non-pathogen is a superior competitor.

### Analysis

**Figure 1:**
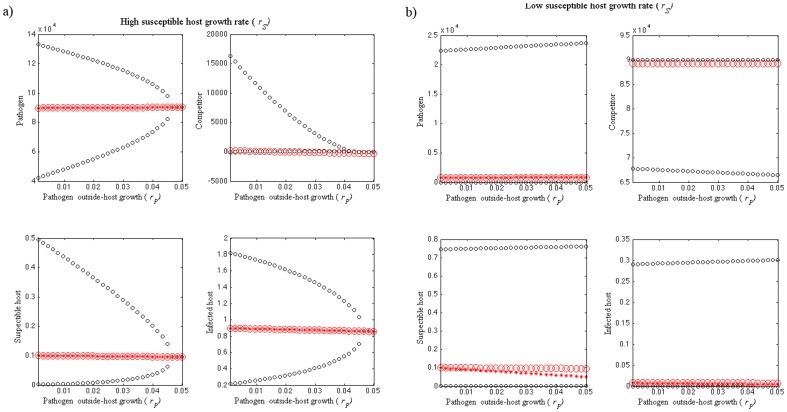
Bifurcation figures of the S-I-P-B dynamics, presenting maximum and minimum values (black circles), as well as equilibrium densities (red stars for when competitor (*B*) is not present, red circles when all the populations are present) of susceptible host (*S*), pathogen (*P*) and non-pathogenic (*B*) population densities in different combinations of outside-host growth rate of pathogen (*r_P_*) parameter values (*r_P_* = 0.001–0.5). a) When susceptible host growth rate (*r_S_*) is high (*r_S_* = 1), increasing *r_P_* stabilizes the disease dynamics. b) Disease dynamics are cyclic when susceptible host growth rate (*r_S_*) is low (*r_S_* = 0.01). Used parameter values are shown in Table 2.

Three different behaviors of the models are analyzed here: invasion of the pathogen where there is no disease present in the beginning. Furthermore, two cases of coexistence may occur, one where the pathogen and competitor coexist and one where the competitor is extinct. The equilibrium population densities are shown in Appendix S1. Coexistence equilibrium population densities are shown in Figures 1a and 1b, where competition coefficients are equal.

#### Invasion analyses

We analyzed both the invasiveness of the pathogen population and the stability of the ecological dynamics when the pathogenic form competes with the non-pathogenic form. The evolution of pathogenicity was analyzed as follows. Consider an equilibrium community in the absence of the pathogen, that is, *S*, *B*>*0* and *P*, *I* = 0. In order to study the stability of this equilibrium solution we linearized the model and studied the local stability of the corresponding Jacobian matrix (Appendix S2). If the equilibrium solution was locally stable, we concluded that the invasion of the pathogen did not succeed. The competition coefficient (*f_BP_*) was tested against six other model parameters. The parameters were given 100 different evenly distributed values from the value range used.

The equilibrium densities of *S* and *B* in the invasion analyses are assumed to be equal to their disease-free equilibrium densities (when *P* = *I* = 0): 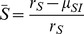
(5)

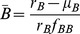
(6)


#### Long-term dynamics of the community

We studied the long-term dynamics in an outside-host competition situation numerically. The simulation length was set to 70 000 days, which was sufficient to uncover the long-term dynamics. Bifurcation diagrams were obtained by scoring the minimum and maximum values of the population fluctuations after removing the initial transient. The outside-host growth rate of the pathogen (*r_P_*), the competition coefficient (*f_BP_*) or the growth rate of the susceptible host (*r_S_*) was varied in bifurcation diagrams, with 30 different evenly distributed values from the value range used The numerical analysis of the model was performed with MATLAB v. 2012b ODE45 solver. The types of the bifurcations, when a locally stable solution turns into a periodic trajectory, or opposite around, were studied by analyzing the eigenvalues of the linearized model around the bifurcation point.

## Results

### Invasion analyses

**Figure 2:**
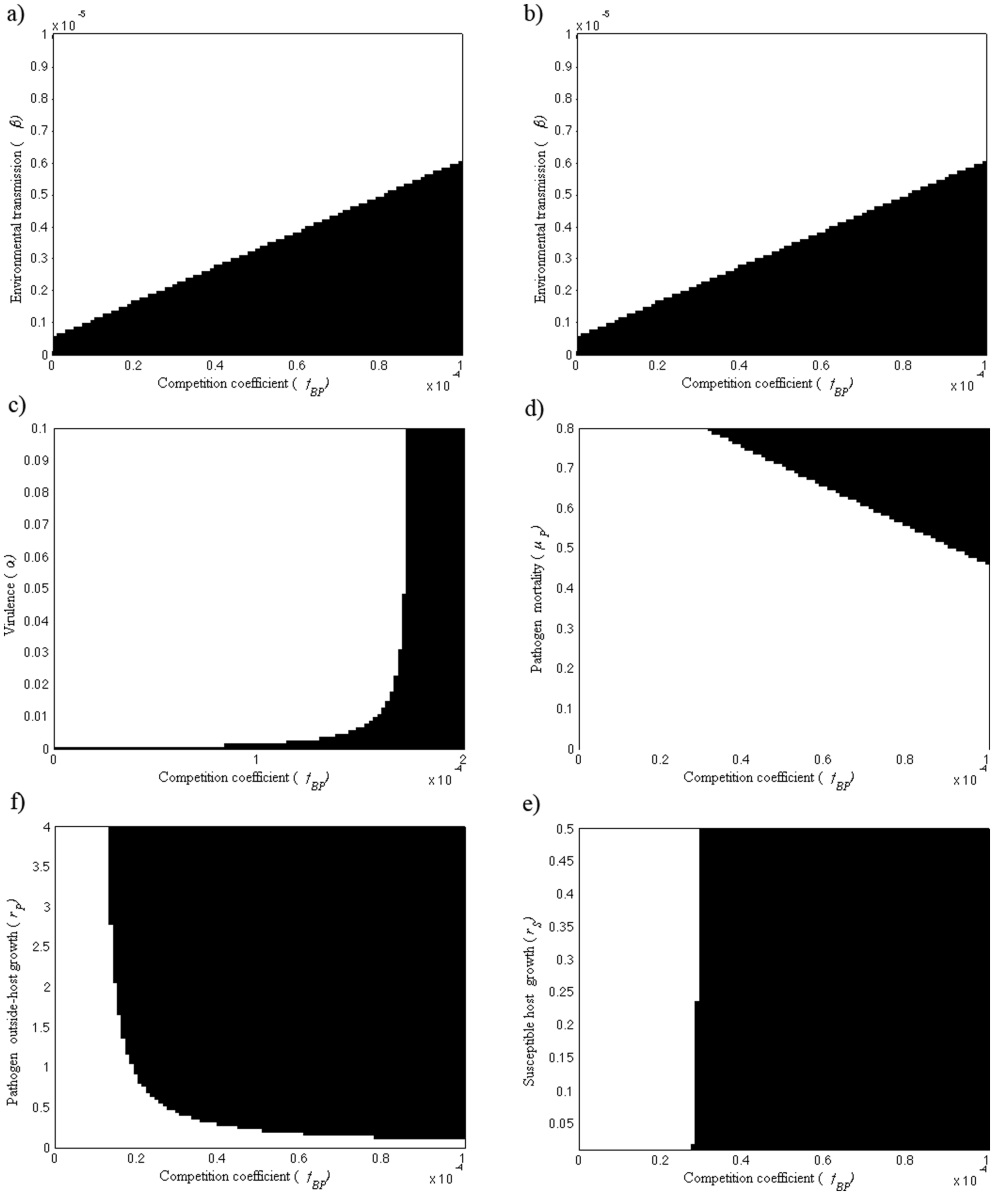
Invasion analyses of a novel environmentally growing opportunist pathogen under outside-host competition situation in different combinations of the competition coefficient (*f_BP_*) parameter values and a) environmental transmission rate (*β*), b) release rate (*Λ*), c) virulence (α), d) pathogen mortality outside-host (*μ_P_*), e) outside-host growth rate of pathogen (*r_P_*) and f) susceptible host growth rate (*r_S_*). The parameter values used are shown in Table 1. The black area shows the parameter combinations for which the equilibrium dynamics are locally stable preventing the invasion of the pathogen. The white area shows where the dynamics become unstable enabling invasion of the new environmentally growing opportunist pathogen (*P*).

As the competition coefficient (*f_BP_*) increases, higher environmental transmission rate (β) and release rate (Λ) are needed in order for a novel environmentally growing opportunist pathogen to invade (Fig. 2a and 2b). Similarly, higher virulence (α) is needed for invasion as *f_BP_* increases, but once *f_BP_* is very high, the pathogen cannot invade (Fig. 2c). High pathogen mortality (μ*_P_*) and high competition coefficient (*f_BP_*) prevent invasion of a novel environmentally growing opportunist pathogen. Otherwise the pathogen is able to invade in the limits of used parameter values (Fig. 2d). Interestingly, higher outside-host growth rate of pathogen (*r_P_*) also prevents invasion of a novel environmentally growing opportunist pathogen as *f_BP_* increases (Fig. 2e). Novel environmentally growing opportunist pathogen is able to invade in lower *f_BP_* values independently of susceptible host growth rate (*r_S_*) but as *f_BP_* increases, higher *r_S_* is needed in order for a novel environmentally growing opportunist pathogen to invade. Once *f_BP_* exceeds 3×10^−4^, the pathogen cannot invade (Fig. 2f). All in all, high competition outside-host limits invasion of a novel environmentally growing opportunist pathogen, while higher inside-host growth promotes invasion of the pathogen.

### Long-term dynamics

#### Pathogen outside-host growth and susceptible host growth

**Figure 3:**
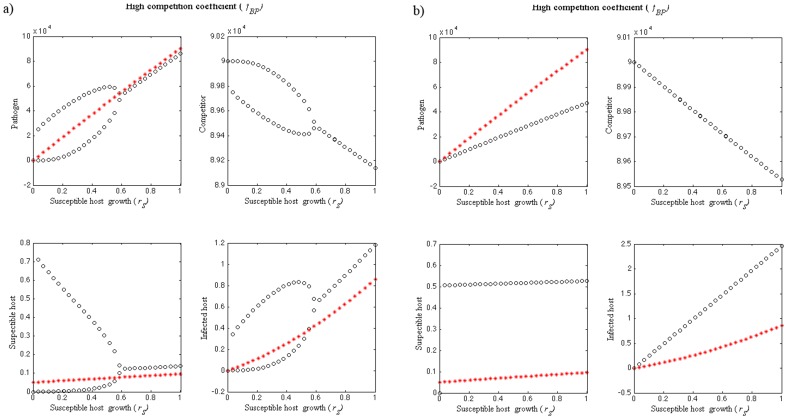
Bifurcation figures of the S-I-P-B dynamics, presenting maximum and minimum values (black circles), as well as equilibrium densities when competitor (*B*) is not present (red stars) of susceptible host (*S*), pathogen (*P*) and non-pathogenic (*B*) population densities in different combinations of susceptible host growth rate of pathogen (*r_S_*) parameter values (*r_S_* = 0–1). In all the figures *f_PB_* = 10^−7^. a) When outside-host growth rate of pathogen (*r_P_*) is 0.05 and the competition coefficient (*f_BP_*) is 10^−5^, decreasing *r_S_* destabilizes the disease dynamics. b) When *r_P_* is (0.05) and *f_BP_* is higher (10^−4^), pathogen population is able to increase and the dynamics are locally stable and all four populations coexist.

**Figure 4:**
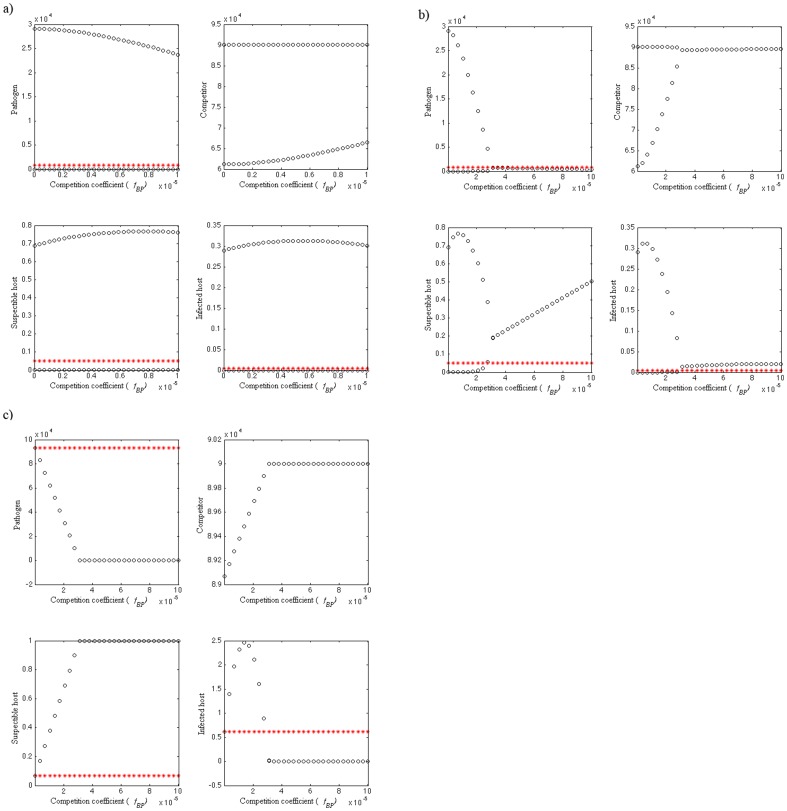
Bifurcation figures of the S-I-P-B dynamics, presenting maximum and minimum values (black circles), as well as equilibrium densities when competitor (*B*) is not present (red stars) of susceptible host (*S*), pathogen (*P*) and non-pathogenic (*B*) population densities in different combinations of the competition coefficient (*f_BP_*) parameter values. a) Dynamics are cyclic when susceptible host growth rate (*r_S_*) is 0.01, outside-host growth rate of pathogen (*r_P_*) is 0.05 and *f_BP_* varies between 0 and 10^−5^. b) When *r_S_* = 0.01, *r_P_* = 0.05 and *f_BP_* = 10^−7^–10^−4^, dynamics are first cyclic and the densities of *P*, *S* and *I* are decreasing as *f_BP_* and the density of *B* increase. As *f_BP_* increases further, pathogen population stabilizes close to zero and the density of *S* starts to increase. c) When *r_S_* = 1, *r_P_* = 0.5, *f_PB_* = 10^−7^ and *f_BP_* = 10^−8^–10^−4^, the coexistence dynamics are locally stable. As *f_BP_* increases, pathogen goes extinct. The parameter values used are shown in Table 2.

Increasing pathogen growth rate outside-host (*r_P_*) has a stabilizing effect on disease dynamics (Fig. 1a where *r_P_* varies between 0.001–0.5, and in Fig. 3a where *r_P_* = 0.05). In Figure 1a, the disease dynamics are periodic when *r_P_* is <0.04. For *r_P_>*0.04 the dynamics stabilize as the non-pathogen goes extinct showing Hopf bifurcation. Similarly, disease dynamics are cyclic when susceptible host growth rate is low (*r_S_* = 0.01, Fig. 1b). Increasing susceptible host growth rate (*r_S_*) has a stabilizing effect on disease dynamics. Hopf bifurcation occurs when the dynamics stabilize (Fig. 3a). As both lower *r_P_* and *r_S_* have a destabilizing effect on the disease dynamics, it is not surprising that *S-I-P-B* dynamics are cyclic when both *r_S_* and *r_P_* are low (Fig. 4a where *r_S_* = 0.01 and *r_P_* = 0.05). On the other hand, stable dynamics can occur also on low *r_S_* and *r_P_* levels depending on the strength of the outside-host competition. When *r_P_* is low (*r_P_* = 0.05) and *f_BP_* exceeds both *f_PB_* and *f_PP_* (*f_BP_* = 10^−4^, *f_PP_* = 10^−5^ and *f_PB_* = 10^−7^), the pathogen population is able to increase as *r_S_* increases and the four populations coexist as long as *r_S_* is positive (Fig. 3b).

#### Outside-host competition

When all the competition coefficient are equal (*f_BP_* = *f_PB_ = f_BB_* = *f_PP_ = *10^−5^) and both *r_S_* and *r_P_* are high (*r_S_* = 1 and *r_P_* = 0.5) the disease dynamics are locally stable and the pathogen drives the non-pathogen to extinction (Fig. 1a). When *r_S_* and *r_P_* are low (*r_S_* = 0.01, *r_P_* = 0.05), the dynamics are first cyclic and the densities of *P*, *S* and *I* are decreasing as *f_BP_* and the density of *B* is increasing (Fig. 4b). Once *f_BP_* increases further, the pathogen population stabilizes close to zero and *S* starts to increase. At this point Hopf bifurcation occurs (Fig. 4b). When *r_S_* and *r_P_* are high and *f_PB_* low (*r_S_* = 1, *r_P_* = 0.5, *f_PB_* = 10^−7^), the dynamics are locally stable (Fig. 4c) For lower values of *f_BP_*, the pathogen is able to infect hosts but its equilibrium value is decreasing with increasing competition. As *f_BP_* exceeds 3×10^−5^, pathogen goes extinct (Fig. 4c).

### Equilibrium densities in the absence of outside-host competition

The equilibrium densities in the absence of outside-host competitor are the same as when the competitor is present in Figure 1a. Otherwise, the equilibrium density of the pathogens is higher and the equilibrium densities of susceptible hosts are lower when the outside-host competitor in absent (Fig. 3a, 3b and 4b) as compared to the population densities when competitor is present and the disease dynamics are locally stable. The equilibrium density of infected host on the other hand is lower when pathogen outside-host growth (*r_P_*) is low (Fig. 3a, 3b and 4b) or higher when *r_P_* is high (Fig. 4c) in the situations where a competitor is absent as compared to the infected host population density when a competitor is present and the disease dynamics are locally stable. In the situation where outside-host competition would result the extinction of the pathogen and infected host population leaving the competitor and susceptible host populations present, removing the competitor leads instead to susceptible host extinction (Fig. 4c).

### Host resource competition

Adding resource competition between susceptible and infected hosts did not alter invasion analysis results (results not shown). Regarding long-term dynamics, resource competition between susceptible and infected hosts lowers the density of the susceptible host and thus pathogen maximal densities in most cases but does not otherwise affect the disease dynamics. An exception to this is the situation in Fig. 1a, where outside-host growth (*r_P_*) varies between 0.001–0.5 and susceptible host growth rate is high (*r_S_* = 0.1). In this case resource competition between susceptible and infected hosts has a stabilizing effect on the disease dynamics and pathogen population density increases as *r_P_* increases while densities of *B*, *S* and *I* decrease (Fig. S2).

### Invasion analysis in other model modifications

It is of general interest how sensitive our model and analyses are with respect to modifications such as including direct transmission, recovery of infected, continuous release of pathogens, sterilization of the hosts instead of immediate death, or resource competition between susceptible and infected hosts. We next review these results.

Adding direct transmission between the hosts (*γ* = 10^−5^) to the model does not influence the invasion analysis results as already shown in the Figure S1.

When the recovery of the infected hosts is possible (recovery rate  = 0.05), higher level of environmental transmission (β) or release (Λ) from infected is needed in order to invade successfully (Fig. S3a, b). Also lower competition coefficient (*f_BP_*) as compared to the situation where infected hosts are unable to recover from the disease prevents invasion of a novel pathogen regardless of the level of virulence (α). Pathogen invasion is still possible when competition coefficient is low enough but the virulence is higher, as compared to the situation where recovery of the infected is not possible (Fig. S3c). Furthermore, lower mortality of the pathogens in the outside-host environment (μ*_P_*) prevents invasion as compared to the situation where infected hosts are unable to recover from the disease (Fig. S3d). Similarly, lower values of pathogen outside-host growth are able to prevent invasion as compared to the model without recovery from infection (Fig. S3e). Invasion likelihood is the same with or without recovery when susceptible host growth is varied (*r_S_*) (Fig. S3f).

When pathogens are released continuously as compared to release during infected host death, the invasion likelihood is the same in all the invasion analysis as when novel pathogens are released as the infected hosts die. An exception to this occurs when the level of virulence (α) is varied such that invasion is successful unless both competition coefficient (*f_BP_*) and α are high (Fig. S4).

When the pathogen sterilizes the infected host but does not cause any extra mortality, the novel pathogen invades in all cases as compared to when the pathogen is virulent. Trivial exception of this is when either the release rate (Λ) or transmission rate (β) are very small.

## Discussion

Traditional epidemiological models (*SI or SIR*) have not considered pathogens that replicate in the outside-host environment where they are also influenced by environmental interactions, such as competition by other species or non-pathogenic forms of the same species. Here we demonstrate that competition in the outside-host environment has a profound effect on disease dynamics and the evolution of pathogenicity. Environmentally growing opportunist pathogen can escape competition within-host and out-compete superior competitors in the outside-host environment by using within-host growth as an additional replication strategy. Thus, novel pathogens can evolve if the fitness advantage due to within-host growth exceeds the cost of virulence traits when competing with the non-pathogenic competitors in the outside-host environment. Strong competition on the other hand may prevent the invasion of novel pathogen or drive the existing environmental pathogen to extinction. The model can produce cyclic and locally stable environmentally growing opportunist disease dynamics depending on the outside-host competition.

According to the coincidental virulence theory, pathogenicity emerges if traits that improve competition ability or defense against predators in the outside-host environment are selected for, and this also coincidentally allow pathogenicity [16], [17]. In contrast, we wanted to investigate whether environmentally growing opportunist pathogenicity could evolve when there is a trade-off between the capability to invade and live within-host, and the efficiency of using outside-host resources for growth or survival. This line of thinking is supported by the existing experimental work [11]. Even assuming this trade-off, our analyses demonstrate that evolution can promote environmentally growing opportunist pathogenicity, when competition in the outside-host environment is not too strong. This result emerges as the environmentally growing opportunist has potentially more resources available when the saprotrophic resources in the outside-host environment are combined with resources gained within-host, as compared to non-pathogenic microbes or obligatory pathogens. On the other hand, faster environmental pathogen outside-host growth prevents invasion when competition against superior non-pathogenic strain is intense. Fast outside-host growth might thus subject novel environmental pathogens to competition and while slower outside-host growth might ensure sustainable infection level of susceptible hosts ensuring within-host growth of a novel environmental pathogen. Selection could also favor higher virulence in an environmentally growing opportunist pathogen as compared to obligatory pathogen as the increase in virulence, pathogen release rate and transmission rate promote invasion of a new environmentally growing opportunist. Increased pathogen release rate also gives competitive advance for new environmentally growing opportunists against non-pathogenic competitors in the outside-host environment. We refrain from arguing that the environmentally growing opportunist pathogenicity could not develop also via coincidental evolution when a pathogenic trait gives an advantage in living in the outside-host environment. In that case the invasion of environmentally growing opportunist pathogen would be trivial.

Strong competition in the outside-host environment may also lead to an extinction of already established highly virulent environmentally growing opportunist pathogen population when they are not able to compensate lower competitive ability with within-host replication even as they are able to otherwise replicate in the outside-host environment in the absence of host. Outside-host competition can thus prevent disease outbreaks. Godfray et al. [33] obtained similar results with their model describing short-term and density-independent dynamics. We found that under some conditions pathogen and non-pathogen can co-exist and disease dynamics are locally stable. We also found that the decreased pathogen outside-host growth has a destabilizing effect on disease dynamics, while increased susceptible host growth rate can stabilize the disease dynamics.

In traditional *SI*-models pathogen growth rate within-host functions as a stabilizing factor as it constrains susceptible host reproduction [45]. Here it is established that low outside-host growth of the pathogen is a destabilizing factor. It is possible that lower *r_P_* allows susceptible hosts to grow periodically by creating cyclic disease dynamics. Lower *r_P_* might also enable survival of an environmentally growing opportunist pathogen faced with strong outside-host competition through stable growth within-hosts by allowing periodical growth of susceptible hosts.

Increasing competition in the outside-host environment at the expense of an environmentally growing opportunistic pathogen may prevent epidemics by preventing the invasion of a pathogenic mutant (i.e. the emergence of a novel disease) and by suppressing the densities of existing pathogen populations. Therefore, our model could be applied to biological control, with the intention of removing highly virulent environmentally growing opportunist pathogens naturally by introducing a superior non-pathogenic competitor in to the outside-host environment or otherwise suppressing the availability of, for example, saprotrophic resources in the outside-host environment. This kind of biological control could for example be propitious in the case of saprotrophic *F. columnare* fish pathogen that is increasingly found in freshwater fish farms. *Flavobacteria* gains extra benefits from life in the fish tank due to high concentration of outside-host resources and high density of potential or already decomposing hosts that act as hotspots for *Flavobacteria* resources. These conditions are probably driving the invasion of novel highly virulent strains of *F. columnare* [46]. Higher virulence is also related to higher within-host growth in saprotrophic *F. columnare* fish pathogen, as pathogen propagation is larger in a dead host [12], promoting higher virulence in saprotrophic opportunist pathogens as compared to other pathogens. Killing the host faster also removes the effect of an immune system so infected hosts do not have time to recover from the infection. For environmentally growing opportunist fish bacteria from the *Flavobacterium* genus the increased and the massive use of antibiotics has brought negative side effects, such as more severe disease symptoms [37]. Environmentally growing opportunist pathogens are also able to escape antibiotic treatment to the outside-host environment. Antibiotic treatment is effective only when fish consume the medication with food. Killing the infected hosts quickly or increasing the necrosis of gill tissue prohibits effects of the antibiotic treatment without a cost to the pathogen that utilizes also the dead host material for its growth. Increased mortality due to necrosis of gill tissue has actually been rising since the antibiotic treatments against columnaris disease were initialized [37]. Thus, efficient methods for the treatment of environmentally growing opportunist diseases are needed especially in conditions where density-suppressing ecological interactions, such as outside-host competition, are relaxed. The conditions described above are also very common in intensive agriculture and farming in general, i.e. low diversity of competitors and excessive nutrients in the outside-host environment that lessen the pathogen trade-off between resource acquisition traits and traits required to invade and resist host immune system.

### Other model versions

When the recovery of infected hosts is possible and pathogens are released as infected hosts die due to the infection, the recovery of the host is a dead-end for the pathogen. Thus, in order to invade successfully under outside-host competition situation higher transmission rate and release rate of a novel pathogen are needed. Also, a higher level of virulence is promoting successful invasion of a novel pathogens as compared to a situation where infected hosts are not able to recover. Yet, lower level outside-host competition is able to prevent the invasion, as pathogens are not able to gain competitive advantage from within-host growth for successful invasion as compared to the situation where infected hosts are unable to recover. Lower outside-host growth is also more beneficial for successful invasion of novel pathogens when infected hosts are able to recover promoting a more obligatory life-style as compared to the situation where the hosts are unable to recover from infection.

When pathogens are released continuously as compared to release upon infected host death, high virulence under high outside-host competition prevents invasion of novel pathogens. Virulence hinders within-host growth in pathogens that are only released from living hosts and thus decreases their ability to compete with superior non-pathogenic competitor in the outside-host environment. Thus, a lower level of virulence would be more beneficial for successful invasion of novel pathogens that are released only from living hosts. Therefore, more benign pathogens that are released continuously from living hosts and do not cause any extra mortality to their hosts are able to invade successfully regardless of the outside-host competition.

## Conclusions

Our model results suggest that a strong competition between a superior non-pathogenic microbe and an environmentally growing opportunist pathogen in the outside-host environment can prevent the invasion of a novel pathogen. Competition can also prevent long-term disease outbreaks by eradicating an existing pathogen population from the outside-host environment.

## Supporting Information

Figure S1 **Direct transmission. In both figures a and b the following parameter values are used:** ***μ_SI_*** **= 0.001, α = 0.1,** ***μ_P_*** **= 0.1,** ***μ_B_*** **= 0.1,** ***β*** **= 10^−5^,** ***Λ*** **= 10^5^,** ***f_PP_*** **= 10^−5^,** ***f_BB_*** **= 10^−5^ and** ***f_PB_*** **= 10^−5^.** a) Invasion analyses of a novel environmentally growing opportunist pathogen under outside-host competition situation in different combinations of the competition coefficient (*f_BP_*) parameter values and direct transmission rate (*γ*). *f_BP_* = 0–2×10^−4^, *γ*  = 0-10^−5^, *r_S_* = 0.01, *r_P_* = 0.05 and *r_B_* = 5. The black area shows in which parameter combinations the dynamics are stable enabling existence of only susceptible host (*S*) and non-pathogenic strain (*B*). The white area shows where the dynamics become unstable enabling invasion of the new environmentally growing opportunist pathogen (*P*). Invasion depends on value of *f_BP_*, independently of the value of *γ*. b) Bifurcation figures of the S-I-P-B dynamics, presenting maximum and minimum values of susceptible host (*S*), infected host (*I*), pathogen (*P*) and non-pathogenic (*B*) population densities in different combinations of direct transmission (*γ*) parameter values (*γ* = 0 to 10^−3^). When susceptible host growth rate of pathogen (*r_S_*) = 0.01 and outside-host growth rate of pathogen (*r_P_*) = 0.05, dynamics are cyclic, but *γ* does not influence the disease dynamics. Other parameter values used: *r_B_* = 1 and *f_BP_* = 10^−5^. For the model, see Supplement S1.
(TIF)

Figure S2 **Bifurcation figures of the S-I-P-B dynamics, presenting maximum and minimum values of susceptible host (** ***S*** **), pathogen (** ***P*** **) and non-pathogenic (** ***B*** **) population densities in different combinations of outside-host growth rate of pathogen (** ***r_P_*** **) parameter values (** ***r_P_*** **= 0.001–0.5) when resource competition between susceptible and infected hosts is considered.** Parameter values are the same as in Fig. 1a. Host carrying capacity (G) is set to 1. For the model, see Supplement S2.
(TIF)

Figure S3 **Invasion analyses of a novel environmentally growing opportunist pathogen when infected hosts are able to recover from infection (** ***r*** **= 0.05).** Parameter values are the same as in Figure 1 a–f (Table 1). Figures show invasion possibility under different competition coefficient (*f_BP_*) parameter values and different parameter values of a) environmental transmission rate (β), b) release rate (Λ), c) virulence (α), d) pathogen mortality outside-host (μ*_P_*), e) outside-host growth rate of pathogen (*r_P_*) and f) susceptible host growth rate (*r_S_*). The black area shows in which parameter combinations the dynamics are locally stable enabling existence of only susceptible host (*S*) and non-pathogenic strain (*B*). The white area shows where the dynamics become unstable enabling invasion of the new environmentally growing opportunist pathogen (*P*). For the model, see Supplement S3.
(TIF)

Figure S4 **Invasion analyses of a novel environmentally growing opportunist pathogen under different competition coefficient (** ***f_BP_*** **) parameter values and virulence (α) values when novel pathogens are release continuously.** Release rate (Λ) is as 10^4^, while other parameter values are similar as in Figure 1c (Table 1). The black area shows in which parameter combinations the dynamics are locally stable enabling existence of only susceptible host (*S*) and non-pathogenic strain (*B*). The white area shows where the dynamics become unstable enabling invasion of the new environmentally growing opportunist pathogen (*P*). For the model, see Supplement S4.
(TIF)

Appendix S1 **Equilibrium population densities.**
(DOCX)

Appendix S2 ***S-I-P-B*** **model linearization and Jacobian matrix.**
(DOCX)

Supplement S1 ***S-I-P-B*** **model when direct transmission (** ***γ*** **) is considered.**
(DOCX)

Supplement S2 ***S-I-P-B*** **model when resource competition between susceptible and infected hosts is considered.**
(DOCX)

Supplement S3 ***S-I-P-B*** **model when recovery of infected hosts (** ***r*** **) is considered.**
(DOCX)

Supplement S4 ***S-I-P-B*** **model when continues release of novel pathogens from the infected hosts is considered.**
(DOCX)

Supplement S5 ***S-I-P-B*** **model when infection sterilizes but causes no extra mortality for the infected hosts.**
(DOCX)
