# Supplementary figures and images for: Invasion Ability and Disease Dynamics of Environmentally Growing Opportunistic Pathogens under Outside-Host Competition
Source: PLoS One. 2014 Nov 21;9(11):e113436. doi: 10.1371/journal.pone.0113436 (PMC4240615; doi:10.1371/journal.pone.0113436)

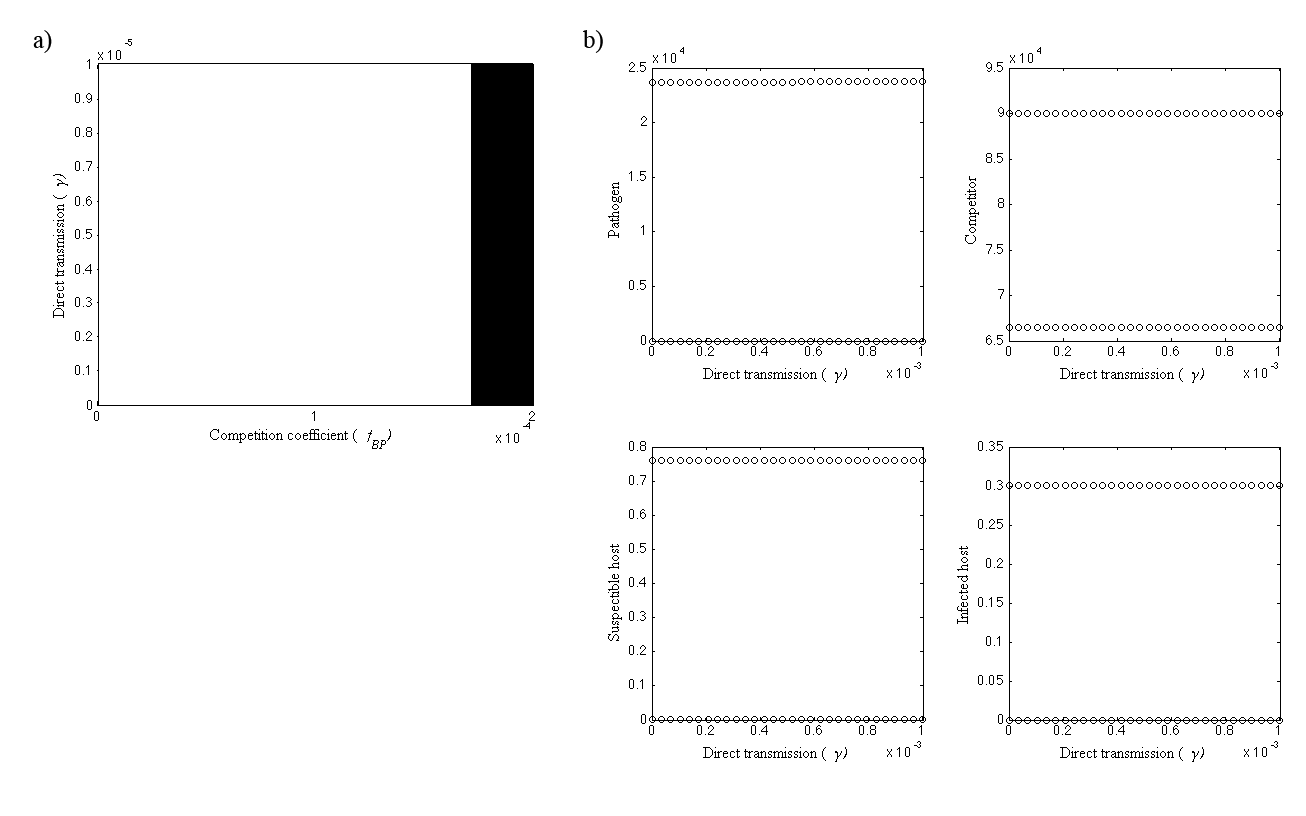

Supplement: Figure S1 — Direct transmission. In both figures a and b the following parameter values are used: μSI = 0.001, α = 0.1, μP = 0.1, μB = 0.1, β = 10−5, Λ = 105, fPP = 10−5, fBB = 10−5 and fPB = 10−5. a) Invasion analyses of a novel environmentally growing opportunist pathogen under outside-host competition situation in different combinations of the competition coefficient (fBP) parameter values and direct transmission rate (γ). fBP = 0–2×10^−4^, γ = 0-10−5, rS = 0.01, rP = 0.05 and rB = 5. The black area shows in which parameter combinations the dynamics are stable enabling existence of only susceptible host (S) and non-pathogenic strain (B). The white area shows where the dynamics become unstable enabling invasion of the new environmentally growing opportunist pathogen (P). Invasion depends on value of fBP, independently of the value of γ. b) Bifurcation figures of the S-I-P-B dynamics, presenting maximum and minimum values of susceptible host (S), infected host (I), pathogen (P) and non-pathogenic (B) population densities in different combinations of direct transmission (γ) parameter values (γ = 0 to 10−3). When susceptible host growth rate of pathogen (rS) = 0.01 and outside-host growth rate of pathogen (rP) = 0.05, dynamics are cyclic, but γ does not influence the disease dynamics. Other parameter values used: rB = 1 and fBP = 10−5. For the model, see Supplement S1. (TIF) [file pone.0113436.s001.tif]

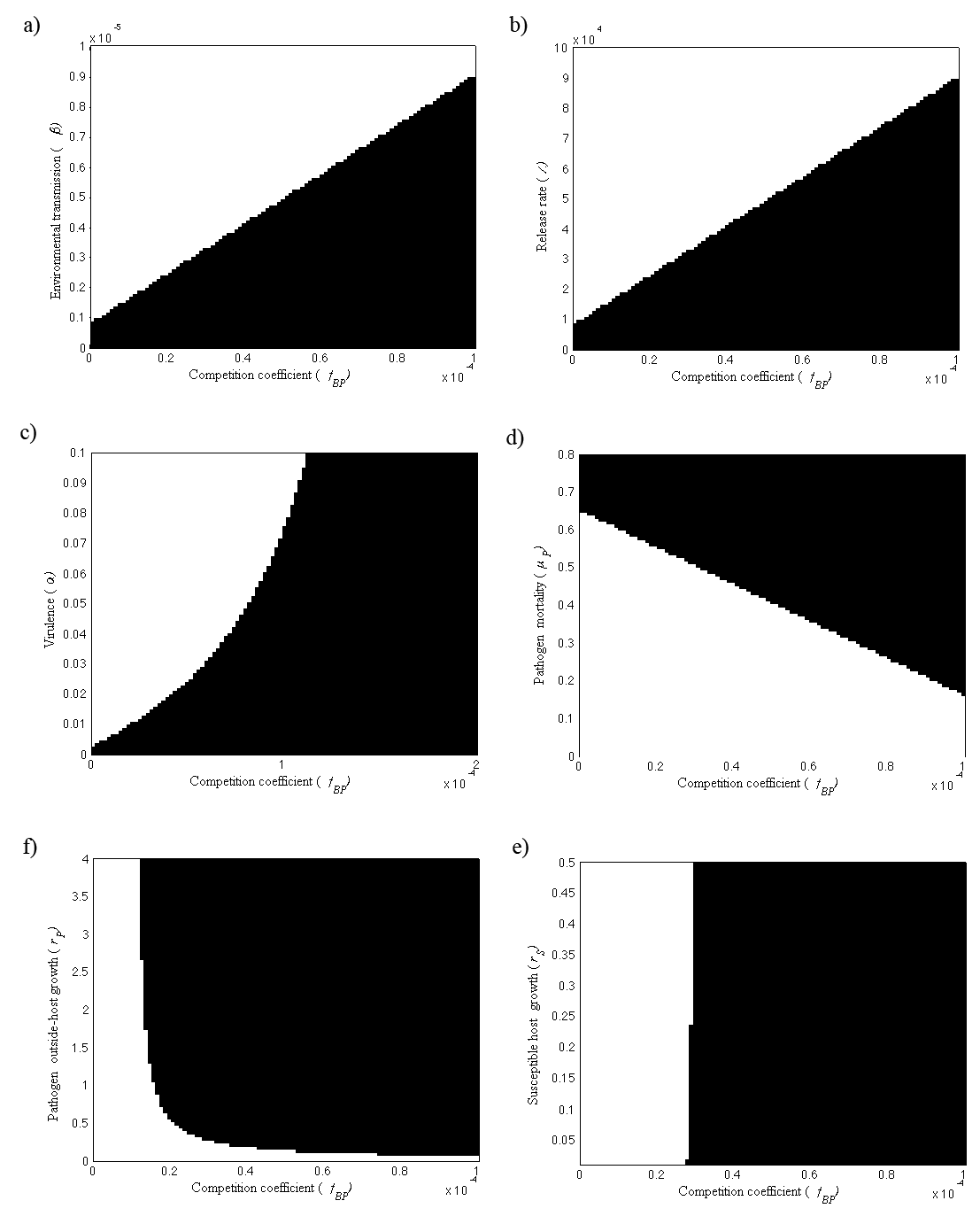

Supplement: Figure S3 — Invasion analyses of a novel environmentally growing opportunist pathogen when infected hosts are able to recover from infection ( r = 0.05). Parameter values are the same as in Figure 1 a–f (Table 1). Figures show invasion possibility under different competition coefficient (fBP) parameter values and different parameter values of a) environmental transmission rate (β), b) release rate (Λ), c) virulence (α), d) pathogen mortality outside-host (μP), e) outside-host growth rate of pathogen (rP) and f) susceptible host growth rate (rS). The black area shows in which parameter combinations the dynamics are locally stable enabling existence of only susceptible host (S) and non-pathogenic strain (B). The white area shows where the dynamics become unstable enabling invasion of the new environmentally growing opportunist pathogen (P). For the model, see Supplement S3. (TIF) [file pone.0113436.s003.tif]
