# Supplementary material for: Invasion Ability and Disease Dynamics of Environmentally Growing Opportunistic Pathogens under Outside-Host Competition
Source: PLoS One. 2014 Nov 21;9(11):e113436. doi: 10.1371/journal.pone.0113436 (PMC4240615; doi:10.1371/journal.pone.0113436)
Supplement: Appendix S1 — Equilibrium population densities. (DOCX) [file pone.0113436.s005.docx]

**Appendix S1.** Equilibrium population densities.

Coexistence equilibrium where $\overline{I}$>0, $\overline{P}$>0, $\overline{S}$>0, $\overline{B}$>0 and when *f_PP_=f_BB_= f_BP_ = f_PB_*

$\begin{matrix} \overline{S}=\frac{\alpha-\mu_{SI}}{\Lambda\alpha\beta}(\mu_{P}-(\frac{r_{P}}{r_{B}})\mu_{B}) \\ \overline{I}=\frac{\beta\overline{S}\overline{P}}{(\alpha+\mu_{SI})} \\ \overline{P}=\frac{r_{S}-\mu_{SI}-r_{S}\overline{S}}{\beta} \\ \overline{B}=\frac{r_{B}(1-f_{PB}\overline{P})-\mu_{B}}{r_{B}f_{BB}} \end{matrix}$

Equilibrium population densities where $\overline{I}$>0, $\overline{P}$>0, $\overline{S}$>0, $\overline{B}$=0

$$\begin{matrix} \overline{S}=\frac{-r_{P}(\frac{1-f_{PP}(r_{S}-\mu_{SI})}{\beta})+\mu_{P}}{(\frac{\Lambda\alpha\beta}{\alpha+\mu_{SI}})+(\frac{r_{P}f_{PP}r_{S}}{\beta})} \\ \overline{I}=\frac{\beta\overline{S}\overline{P}}{(\alpha+\mu_{SI})} \\ \overline{P}=\frac{r_{S}-\mu_{SI}-r_{S}\overline{S}}{\beta} \end{matrix}$$
