# Supplementary material for: Invasion Ability and Disease Dynamics of Environmentally Growing Opportunistic Pathogens under Outside-Host Competition
Source: PLoS One. 2014 Nov 21;9(11):e113436. doi: 10.1371/journal.pone.0113436 (PMC4240615; doi:10.1371/journal.pone.0113436)
Supplement: Appendix S2 — S-I-P-B model linearization and Jacobian matrix. (DOCX) [file pone.0113436.s006.docx]

**Appendix S2.** *S-I-P-B* model linearization and Jacobian matrix.

Linearization:

$$\begin{matrix} \frac{ds}{dt}=r_{S}s(t)-r_{S}2\overline{S}s(t)-\beta\overline{S}p(t)-\beta\overline{P}s(t)-\mu_{SI}s(t) \\ \frac{di}{dt}=\beta\overline{S}p(t)+\beta\overline{P}s(t)-\alpha i(t)-\mu_{SI}i(t) \\ \frac{dp}{dt}=\Lambda\alpha i(t)+r_{P}p(t)-r_{P}f_{PP}2\overline{P}p(t)-r_{P}f_{BP}\overline{B}p(t)-r_{P}f_{BP}\overline{P}b(t)-\mu_{P}p(t) \\ \frac{db}{dt}=r_{B}b(t)-r_{B}f_{PB}\overline{P}b(t)-r_{B}f_{PB}\overline{B}p(t)-r_{B}f_{BB}2\overline{B}b(t)-\mu_{B}b(t) \end{matrix}$$

Jacobian matrix at $\overline{I}$>0, $\overline{P}$>0, $\overline{S}$>0, $\overline{B}$>0 (coexistence)

$$\left[\begin{matrix} r_{S}-r_{S}2\overline{S}-\beta\overline{P}-\mu_{SI} & 0 & -\beta\overline{S} & 0 \\ \beta\overline{P} & -\alpha-\mu_{SI} & \beta\overline{S} & 0 \\ 0 & \Lambda\alpha& r_{P}-r_{P}f_{PP}2\overline{P}-r_{P}f_{BP}\overline{B}-\mu_{P} & -r_{P}f_{BP}\overline{P} \\ 0 & 0 & -r_{B}f_{PB}\overline{B} & r_{B}-r_{B}f_{PB}\overline{P}-r_{B}f_{BB}2\overline{B}-\mu_{B} \end{matrix}\right]$$
