# Supplementary material for: Invasion Ability and Disease Dynamics of Environmentally Growing Opportunistic Pathogens under Outside-Host Competition
Source: PLoS One. 2014 Nov 21;9(11):e113436. doi: 10.1371/journal.pone.0113436 (PMC4240615; doi:10.1371/journal.pone.0113436)
Supplement: Supplement S1 — S-I-P-B model when direct transmission ( γ ) is considered. (DOCX) [file pone.0113436.s007.docx]

**Supplement S1**. *S-I-P-B* model when direct transmission (*γ*) is considered.

$$\begin{matrix} \frac{dS}{dt}=r_{S}S\left[1-S\right]-\beta SP-\gamma SI-\mu_{SI}S \\ \frac{dI}{dt}=\beta SP+\gamma SI-\left(\alpha+\mu_{SI}\right)I \\ \frac{dP}{dt}=\Lambda\alpha I+r_{P}(1-f_{PP}P-f_{BP}B)P-\mu_{P}P \\ \frac{dB}{dt}=r_{B}(1-f_{PB}P-f_{BB}B)B-\mu_{B}B \end{matrix}$$

Linearization:

$\begin{matrix} \frac{ds}{dt}=r_{S}s(t)-r_{S}2\overline{S}s(t)-\beta\overline{P}s(t)-\beta\overline{S} p(t)-\mu_{SI}s(t)-\gamma\overline{I}s(t)-\gamma\overline{S}i(t) \\ \frac{di}{dt}=\beta\overline{S}p(t)+\beta\overline{P}s(t)+\gamma\overline{I}s(t)+\gamma\overline{S}i(t)-\alpha i(t)-\mu_{SI}i(t) \\ \frac{dp}{dt}=\Lambda\alpha i(t)+r_{P}p(t)-r_{P}f_{PP}2\overline{P}p(t)-r_{P}f_{BP}\overline{B}p(t)-r_{P}f_{BP}\overline{P}b(t)-\mu_{P}p(t) \\ \frac{db}{dt}=r_{B}b(t)-r_{B}f_{PB}\overline{P}b(t)-r_{B}f_{PB}\overline{B}p(t)-r_{B}f_{BB}2\overline{B}b(t)-\mu_{B}b(t) \end{matrix}$

At $\overline{I}$=0, $\overline{P}$=0, $\overline{S}$>0, $\overline{B}$>0 the Jacobian matrix becomes

$$\left[\begin{matrix} r_{S}-r_{S}2\overline{S}-\mu_{SI} & -\gamma\overline{S} & -\beta\overline{S} & 0 \\ 0 & \gamma\overline{S}-\alpha-\mu_{SI} & \beta\overline{S} & 0 \\ 0 & \Lambda\alpha& r_{P}-r_{P}f_{BP}\overline{B}-\mu_{P} & 0 \\ 0 & 0 & -r_{B}f_{PB}\overline{B} & r_{B}-r_{B}f_{BB}2\overline{B}-\mu_{B} \end{matrix}\right]$$
