# Supplementary material for: Invasion Ability and Disease Dynamics of Environmentally Growing Opportunistic Pathogens under Outside-Host Competition
Source: PLoS One. 2014 Nov 21;9(11):e113436. doi: 10.1371/journal.pone.0113436 (PMC4240615; doi:10.1371/journal.pone.0113436)
Supplement: Supplement S2 — S-I-P-B model when resource competition between susceptible and infected hosts is considered. (DOCX) [file pone.0113436.s008.docx]

**Supplement S2**. *S-I-P-B* model when resource competition between susceptible and infected hosts is considered.

$$\begin{matrix} \frac{dS}{dt}=r_{S}S\left[1-\frac{1}{G}S-\frac{1}{G}I\right]-\beta SP-\mu_{SI}S \\ \frac{dI}{dt}=\beta SP-\left(\alpha+\mu_{SI}\right)I \\ \frac{dP}{dt}=\Lambda\alpha I+r_{P}(1-f_{PP}P-f_{BP}B)P-\mu_{P}P \\ \frac{dB}{dt}=r_{B}(1-f_{PB}P-f_{BB}B)B-\mu_{B}B \end{matrix}$$
