# Supplementary material for: Invasion Ability and Disease Dynamics of Environmentally Growing Opportunistic Pathogens under Outside-Host Competition
Source: PLoS One. 2014 Nov 21;9(11):e113436. doi: 10.1371/journal.pone.0113436 (PMC4240615; doi:10.1371/journal.pone.0113436)
Supplement: Supplement S4 — S-I-P-B model when continues release of novel pathogens from the infected hosts is considered. (DOCX) [file pone.0113436.s010.docx]

**Supplement S4.** *S-I-P-B* model when continues release of novel pathogens from the infected hosts is considered.

***$\begin{matrix} \frac{dS}{dt}=r_{S}S\left[1-S\right]-\beta SP-\mu_{SI}S \\ \frac{dI}{dt}=\beta SP-\left(\alpha+\mu_{SI}\right)I \\ \frac{dP}{dt}=\Lambda I+r_{P}(1-f_{PP}P-f_{BP}B)P-\mu_{P}P \\ \frac{dB}{dt}=r_{B}(1-f_{PB}P-f_{BB}B)B-\mu_{B}B \end{matrix}$***

Linearization:

$$\begin{matrix} \frac{ds}{dt}=r_{S}s(t)-r_{S}2\overline{S}s(t)-\beta\overline{S}p(t)-\beta\overline{P}s(t)-\mu_{SI}s(t) \\ \frac{di}{dt}=\beta\overline{S}p(t)+\beta\overline{P}s(t)-\alpha i(t)-\mu_{SI}i(t) \\ \frac{dp}{dt}=\Lambda i(t)+r_{P}p(t)-r_{P}f_{PP}2\overline{P}p(t)-r_{P}f_{BP}\overline{B}p(t)-r_{P}f_{BP}\overline{P}b(t)-\mu_{P}p(t) \\ \frac{db}{dt}=r_{B}b(t)-r_{B}f_{PB}\overline{P}b(t)-r_{B}f_{PB}\overline{B}p(t)-r_{B}f_{BB}2\overline{B}b(t)-\mu_{B}b(t) \end{matrix}$$

Jacobian matrix at

$$\left[\begin{matrix} r_{S}-r_{S}2\overline{S}-\mu_{SI} & 0 & -\beta\overline{S} & 0 \\ 0 & -\alpha-\mu_{SI} & \beta\overline{S} & 0 \\ 0 & \Lambda& r_{P}-r_{P}f_{BP}\overline{B}-\mu_{P} & 0 \\ 0 & 0 & -r_{B}f_{PB}\overline{B} & r_{B}-r_{B}f_{BB}2\overline{B}-\mu_{B} \end{matrix}\right]$$
